# Fuc‐S, a New Degraded Polysaccharide From Fucoidan, Alleviates Type 2 Diabetes‐Associated Liver Injury Through Modulating Gut Microbiota

**DOI:** 10.1002/fsn3.71321

**Published:** 2026-01-16

**Authors:** Bin Du, Caihong Cheng, Jinxiu Feng, Yue Liang, Wang Tao, Baojun Xu, Jiao Peng, Guangtao Zhang, Yuedong Yang

**Affiliations:** ^1^ Hebei Key Laboratory of Natural Products Activity Components and Function Hebei Normal University of Science and Technology Qinhuangdao China; ^2^ Department of Pharmacy Peking University Shenzhen Hospital Shenzhen Guangdong China; ^3^ Food Science and Technology Program, Department of Life Sciences Beijing Normal‐Hong Kong Baptist University Zhuhai China; ^4^ Department of Hepatobiliary and Pancreatic Surgery Peking University Shenzhen Hospital Shenzhen Guangdong China

**Keywords:** fucoidan, gut microbiota, liver injury, metabolites, T2DM

## Abstract

Type 2 diabetes mellitus (T2DM) is frequently associated with liver injury. This study examines the therapeutic potential of *Fucus vesiculosus* ‐derived low‐molecular‐weight fucoidan (Fuc‐S) in mitigating T2DM‐related hepatic damage. In STZ/HFD‐induced diabetic mice, Fuc‐S treatment (100 or 200 mg/kg, 5 weeks) significantly improved glucose tolerance, lipid metabolism, and liver function, while reducing hepatic steatosis and serum ALT/AST levels. Fuc‐S enhanced hepatic antioxidant defenses, increasing SOD, CAT, and GSH‐Px activity while decreasing MDA levels. Gut microbiota analysis showed that Fuc‐S promoted the growth of beneficial bacteria ( *Bacteroides acidifaciens* ) and elevated fecal short‐chain fatty acids (SCFAs), such as acetate, propionate, and butyrate. Furthermore, Fuc‐S reinforced intestinal barrier integrity by upregulating tight junction proteins (ZO‐1 and occludin). These results indicate that Fuc‐S alleviates T2DM‐induced liver injury by modulating the gut microbiota‐SCFA‐liver axis, thereby reducing oxidative stress and inflammation. The study suggests Fuc‐S as a promising dietary intervention for T2DM, acting through multi‐target microbiota‐metabolite interactions.

## Graphical abstract

Fuc‐S alleviated type 2 diabetes‐associated liver injury through gut microbiota‐SCFA‐liver axis regulation. Fuc‐S treatment significantly improved glucose tolerance, lipid metabolism, and liver function, while reducing hepatic steatosis and serum ALT/AST levels. Fuc‐S reinforced intestinal barrier integrity by upregulating tight junction proteins (ZO‐1 and occludin).

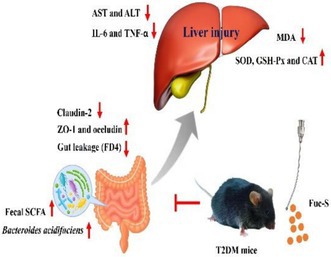

**Abbreviations**
ALT: alanine aminotransferase
AST: aspartate aminotransferase
AUC: area under the curve
CAT: catalase
Con: control group
ELISA: enzyme‐linked immunosorbent assay
FFA: free fatty acid
Fuc‐S:
*Fucus vesiculosus* ‐derived low‐molecular‐weight fucoidan
GSH‐Px: glutathione peroxidase
HDL‐C: high‐density lipoprotein cholesterol
H&E: hematoxylin and eosin
HFD: high‐fat diet
IL‐6: interleukin‐6
LDL‐C: low‐density lipoprotein cholesterol
MDA: malondialdehyde
Met: metformin
Mod: model group
NAFLD: non‐alcoholic fatty liver disease
NASH: non‐alcoholic steatohepatitis
OGTT: oral glucose tolerance test
OTUs: operational taxonomic units
PFA: paraformaldehyde
P‐H: high‐dose fucoidan sulfate group
P‐L: low‐dose fucoidan sulfate group
ROS: reactive oxygen species
SCFAs: short‐chain fatty acids
SOD: superoxide dismutase
STZ: streptozotocin
T2DM: type 2 diabetes mellitus
TC: total cholesterol
TG: triglycerides
TG: triglycerides
TNF‐α: tumor necrosis factor‐alpha

## 1 Introduction

Type 2 Diabetes Mellitus (T2DM) has emerged as a major global public health challenge, with its prevalence rising at an alarming rate (Chen et al. 2025). Growing evidence identifies liver injury as a critical secondary complication of T2DM (Younossi et al. 2016). Notably, 50%–70% of T2DM patients develop non‐alcoholic fatty liver disease (NAFLD), and 10%–30% progress to non‐alcoholic steatohepatitis (NASH), markedly elevating the risks of fibrosis, cirrhosis, and hepatocellular carcinoma (En Li Cho et al. 2023). While current pharmacotherapies—including insulin secretagogues and SGLT‐2 inhibitors—effectively control hyperglycemia, they are associated with adverse effects such as hypoglycemia, gastrointestinal disturbances, and urinary tract infections, while offering minimal hepatic protection (ElSayed et al. 2023). Given these limitations, nutritional interventions, particularly dietary fiber supplementation, have gained prominence due to their favorable safety profile and pleiotropic mechanisms. Dietary fibers exert beneficial effects by modulating the gut microbiota‐liver axis, improving insulin sensitivity, and reducing oxidative stress (Canfora et al. 2019; Reynolds et al. 2020; Weickert and Pfeiffer 2018), positioning them as a promising therapeutic strategy for T2DM management.

Fucoidan, a sulfated fucose‐rich polysaccharide predominantly derived from edible brown algae ( *Fucus vesiculosus* ), demonstrates a broad spectrum of bioactivities closely tied to its unique structural characteristics, including sulfation patterns, glycosidic linkages, and molecular weight distribution (Li et al. 2008). Emerging research highlights its multifaceted therapeutic potential across diverse pathological conditions. Notably, fucoidan has shown promise in mitigating metabolic disorders through mechanisms such as AMPK‐mediated enhancement of insulin sensitivity in muscle tissue, underscoring its antidiabetic properties (Jeong et al. 2013). Its anti‐inflammatory effects are attributed to the modulation of key pathways, including NF‐κB signaling inhibition (Park et al. 2011), while antitumor activity arises from its ability to remodel the tumor microenvironment (Atashrazm et al. 2015). Further investigations reveal its capacity to suppress angiogenesis via VEGF inhibition and exert bidirectional immunomodulatory effects, fine‐tuning immune responses (Fitton et al. 2015; Koyanagi et al. 2003). Collectively, these findings position fucoidan as a versatile bioactive compound with significant translational potential in metabolic, inflammatory, oncologic, and immunologic contexts.

In our previous study, a low‐molecular‐weight Fuc‐S was isolated from *F. vesiculosus* through ultrasonic‐assisted degradation, with its structural properties comprehensively characterized by HPGPC, FT‐IR, and NMR (Xiao et al. 2022). Fuc‐S demonstrated notable enteroprotective effects in a DSS‐induced colitis model, primarily by reshaping gut microbiota composition—particularly through the enrichment of beneficial taxa such as *Akkermansia*—and modulating host‐microbial tryptophan metabolism, leading to elevated levels of immunomodulatory metabolites like indole‐3‐propionic acid. Building upon these findings, the present study aims to elucidate whether Fuc‐S can ameliorate T2DM‐associated liver injury via the “gut microbiota‐metabolite‐liver” axis, with a focus on microbial community restructuring and the regulation of key functional metabolites, including short‐chain fatty acids (SCFAs). By unraveling the underlying mechanisms, this work seeks to provide novel insights into fucoidan's therapeutic potential in T2DM and its hepatic complications, ultimately contributing to the development of precision nutrition strategies that leverage microbiota‐metabolite crosstalk for metabolic health.

## 2 Materials and Methods

### 2.1 Materials

Fucoidan, derived from *F. vesiculosus* , was procured from Shanghai Eysin Biotechnology Co. Ltd. (Shanghai, China). Streptozotocin (STZ), metformin (Met), hematoxylin, and eosin were obtained from Sigma‐Aldrich (St. Louis, MO, USA). A high‐fat diet (HFD; #12492) was supplied by Research Diets Inc. (Bethlehem, PA, USA). Enzyme‐linked immunosorbent assay (ELISA) kits for superoxide dismutase (SOD), catalase (CAT), glutathione peroxidase (GSH‐Px), malondialdehyde (MDA), aspartate aminotransferase (AST), alanine aminotransferase (ALT), triglycerides (TG), low‐density lipoprotein cholesterol (LDL‐C), and high‐density lipoprotein cholesterol (HDL‐C) were purchased from Nanjing Jiancheng Bioengineering Institute (Nanjing, China). ELISA kits for interleukin‐6 (IL‐6) and tumor necrosis factor‐alpha (TNF‐α) were sourced from JiangLai Biology (Shanghai, China). Short‐chain fatty acids (SCFAs), including acetic acid, propionic acid, isobutyric acid, and butyric acid, were acquired from Aladdin Industrial Corporation (Shanghai, China). 2‐Ethylbutyric acid was purchased from Merck KGaA (Darmstadt, Germany). The QIAquick Gel Extraction Kit was obtained from QIAGEN (Hilden, Germany), and the DNA extraction kit was procured from TIANGEN Biotech (Beijing, China).

### 2.2 Preparation and Characterization of Fuc‐S

Fuc‐S was prepared from the brown alga *F. vesiculosus* through a two‐step process, as previously described (Xiao et al. 2022). First, crude fucoidan (purity ≥ 85%) was obtained via water extraction (10:1 mL/g liquid‐to‐material ratio, 2 h reflux) followed by ethanol precipitation (4:1 mL/g alcohol‐to‐sample ratio). This crude fucoidan was then degraded by ultrasonication of a 1 g/L aqueous solution at 950 W and 25 kHz for 5 h in an ice‐water bath. The product, Fuc‐S, was obtained after freeze‐drying. The molecular weight of the resulting Fuc‐S was determined to be 156 kDa by HPSEC‐MALLS, and its monosaccharide composition was glucose: galactose: xylose: rhamnose: mannose: galacturonic acid: glucuronic acid in a molar ratio of 0.49: 0.28: 0.12: 0.01: 0.02: 0.04: 0.01: 0.02.

### 2.3 Animal Studies

The experimental protocol was approved by the Institutional Animal Care and Use Committee of Shenzhen PKU‐HKUST Medical Center (Protocol No. 2021‐791). Male C57BL/6J mice (6–7 weeks old, 21 ± 1 g) were obtained from Beijing Vital River Laboratory Animal Technology Co. Ltd. (Beijing, China; License No. SCXK JING 2021‐0006). After 1 week of acclimatization, eight mice were randomly assigned to the normal control group (Con), while the remaining mice were fed a high‐fat diet for 4 weeks, followed by intraperitoneal injection of streptozotocin (STZ, 25 mg/kg) for three consecutive days to establish a T2DM model, according to a previously established protocol (Zhai et al. 2018). Successful modeling was defined as fasting blood glucose ≥ 11.1 mmol/L (Xiao et al. 2017). Thirty‐two diabetic mice were then randomized into four groups: the model group (Mod), metformin group (Met), low‐dose Fuc‐S group (P‐L), and high‐dose Fuc‐S group (P‐H). Treatments were administered via oral gavage for 5 weeks. The dosage of metformin, used as the positive control, was set at 250 mg/kg, while the doses of P‐L and P‐H were set at 100 and 200 mg/kg, respectively, based on previous studies (Xiao et al. 2022; Zhao et al. 2023).

### 2.4 Metabolic Parameter Assessment

Body weight, food intake, and fasting blood glucose were monitored weekly. An oral glucose tolerance test (OGTT) was performed after a 16‐h fast before the end of the experiment. Mice were administered glucose (2 g/kg) by oral gavage, and blood glucose levels were measured at 0, 30, 60, 90, and 120 min. The area under the curve (AUC) was calculated to evaluate glucose tolerance.

### 2.5 Histopathological Evaluation

Liver and colon tissues were fixed in 4% paraformaldehyde (PFA), embedded in paraffin, sectioned (4–5 μm), and stained with hematoxylin and eosin (H&E). Histopathological evaluation was conducted by blinded observers according to standardized criteria.

### 2.6 Biochemical Analysis

Liver tissues were homogenized in protein extraction buffer containing protease/phosphatase inhibitors. Total protein concentration was determined by BCA assay (Beyotime, Shanghai, China). The levels of SOD, CAT, GSH‐Px, MDA, ALT, AST, and inflammatory cytokines (IL‐6, TNF‐α) in liver tissue, as well as serum AST, ALT, TG, LDL‐C, and HDL‐C levels, were quantified using commercial ELISA kits according to the manufacturer's instructions.

### 2.7 Intestinal Permeability Evaluation

Intestinal permeability was evaluated in vivo using fluorescein isothiocyanate–dextran (FITC‐dextran; 4 kDa; Sigma‐Aldrich, USA) according to established protocols (Huan et al. 2023). Briefly, colitis‐induced mice were fasted for 12 h and subsequently administered FITC‐dextran (0.6 mg/g body weight) via oral gavage prior to experimental termination. Three hours post‐administration, mice were anesthetized, and blood samples were collected via the retro‐orbital plexus for fluorescence spectrophotometric quantification of serum FITC‐dextran levels.

### 2.8 Fecal 16S rRNA Gene Sequencing Analysis

Total DNA was extracted from fecal samples using a DNA extraction kit. DNA quality and concentration were assessed by measuring the absorbance ratios at 260/280 nm and 260/230 nm. The V3–V4 hypervariable region of the 16S rRNA gene was amplified via PCR using specific primers 338F (ACTCCTACGGGAGGCAGCAG) and 806R (GGACTACHVGGGTWTCTAAT). The PCR products were purified using the QIAquick Gel Extraction Kit, quantified by real‐time PCR, and subjected to paired‐end sequencing on the Illumina MiSeq PE300 platform (Illumina Inc., USA). For bioinformatics analysis, raw fastq files were quality‐filtered using Cutadapt (v1.9.1). Operational taxonomic units (OTUs) were clustered at 97% similarity using UPARSE (v7.0.1001), and chimeric sequences were removed with UCHIME. Taxonomic classification was performed using the RDP classifier (http://rdp.cme.msu.edu/) based on the SILVA ribosomal RNA gene database, as previously described (Huan et al. 2023).

### 2.9 SCFA Quantification

Fecal SCFAs (acetate, propionate, and butyrate/isobutyrate) were quantified using a modified version of our previously described method (Peng et al. 2022). Briefly, 100 mg of feces were homogenized in 0.4 mL deionized water, acidified with 0.1 mL 50% H₂SO₄, and spiked with 0.5 mL 2‐ethylbutyric acid (internal standard). After centrifugation (12,000 rpm, 15 min, 4°C), the supernatant was extracted with 0.5 mL diethyl ether and analyzed via GC (Shimadzu GC‐2010 Plus) equipped with an FID and a ZKAT‐624 column (30 m × 0.53 mm × 0.3 μm, Zhongke Antai). Nitrogen carrier gas flow was 6 mL/min (split ratio 10:1). Injection volume: 1 μL; injector/detector temperatures: 300°C. Oven program: 140°C (13.5 min), ramp to 250°C at 120°C/min, hold 5 min. Data were processed using GC Solution software (Shimadzu).

### 2.10 RNA Extraction and RT‐qPCR

Total RNA was isolated from colon tissues using TRIzol reagent (Thermo Fisher, USA). Subsequently, cDNA was synthesized with a reverse transcription kit (TaKaRa, Japan). Quantitative real‐time PCR (RT‐qPCR) was performed on an ABI 7500 system using SYBR Green Master Mix (Roche, Germany). Gene expression levels were normalized to GAPDH and calculated via the 2^−ΔΔCT^ method. The primer sequences used were as follows: mouse ZO‐1 (F: ACCCGAAACTGATGCTGTGGATAG; R: GCTGGCTGGCTGTACTGTGAG), mouse occludin (F: AGGCAGCCTCGGTACAGCAG; R: AGGCAGCCTCGGTACAGCAG); mouse claudin‐2 (F: AGCATTGTGACGGCGGTTGG; R: GGCAGCCTGGATGTCAGCAG); mouse GAPDH (F: CATCACTGCCACCCAGAAGACTG; R: ATGCCAGTGAGCTTCCCGTTCAG).

### 2.11 Western Blot Detection

Proteins were extracted from colon tissues using RIPA buffer (Thermo Scientific, USA) supplemented with protease and phosphatase inhibitors (Roche, Basel, Switzerland). Protein concentrations were determined with a BCA assay kit (Beyotime, Shanghai, China). The samples were then subjected to SDS‐PAGE (Beyotime, Shanghai, China) and transferred onto PVDF membranes. After blocking with 3% BSA, the membranes were probed overnight with primary antibodies against occludin and GAPDH (1:1000; Cell Signaling Technology, USA), followed by incubation with appropriate HRP‐conjugated secondary antibodies (1:2000). The protein bands were visualized using an ECL detection system (Abclonal, China) and quantified with ImageJ software (NIH, USA).

### 2.12 Statistical Analysis

Data were analyzed by one‐way ANOVA followed by Duncan's multiple range test, using GraphPad Prism 6.0. The results are presented as mean ± SEM, with a *p*‐value < 0.05 deemed statistically significant.

## 3 Results

### 3.1 Therapeutic Effects of Fuc‐S on STZ/High‐Fat Diet‐Induced Diabetic Mice

**FIGURE 1:**
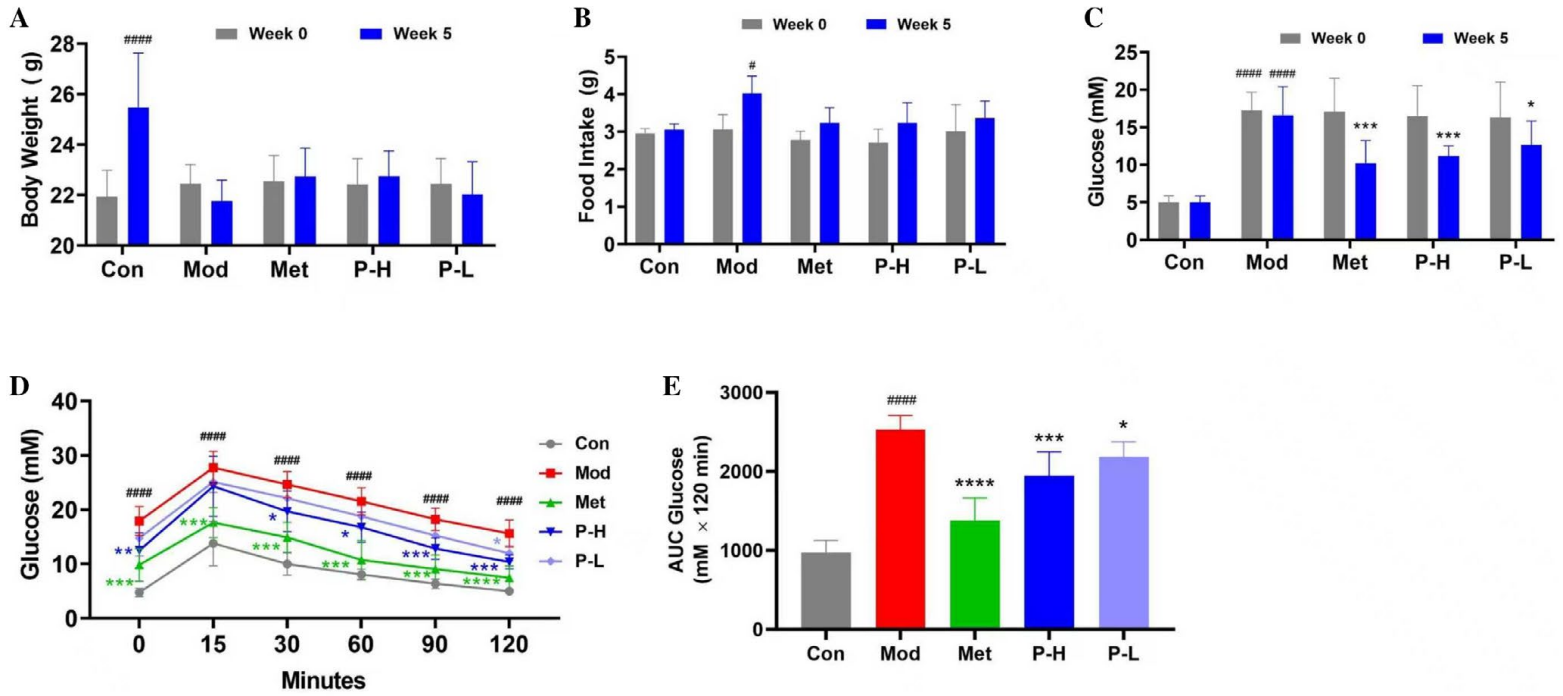
Fuc‐S treatment alleviates STZ/HFD‐induced diabetes in mice. (A) Body weight and (B) food intake at baseline (Week 0) and endpoint (Week 5). (C) Fasting glucose levels at Weeks 0 and 5. (D) OGTT and (E) corresponding AUC at termination. T2DM was induced by 4‐week HFD followed by STZ (25 mg/kg/day i.p., 3 days). Hyperglycemic mice (fasting glucose ≥ 11 mmol/L, 7 days) received Fuc‐S or Met for 5 weeks. Metabolic parameters were monitored weekly (*n* = 8, mean ± SEM). ^#^ *p* < 0.05, ^####^ *p* < 0.001 versus Con; *p < 0.05, ***p < 0.001, ****p < 0.0001 versus Mod. Groups: Con (control), Mod (model), Met (metformin), P‐H/L (high/low‐dose Fuc‐S).

To evaluate the antidiabetic potential of Fuc‐S, a 5‐week oral intervention was conducted in a murine model of T2DM induced by high‐fat diet (HFD) and STZ. While Fuc‐S treatment led to a modest, non‐significant increase in body weight compared to the diabetic model group (Figure 1A), it significantly normalized hyperphagia—a hallmark metabolic disturbance in diabetic mice—comparable to Met (Figure 1B). Notably, Fuc‐S and Met both markedly attenuated hyperglycemia after 5 weeks of treatment (*p* < 0.001 or *p* < 0.05 vs. model group; Figure 1C). OGTT test further confirmed these hypoglycemic effects, demonstrating that Fuc‐S not only improved glucose intolerance but also reduced AUC for blood glucose (Figure 1D,E). Collectively, these findings establish Fuc‐S as an effective glucose‐lowering agent in experimental T2DM.

### 3.2 Fuc‐S Ameliorates Diabetes‐Associated Dyslipidemia

**FIGURE 2:**
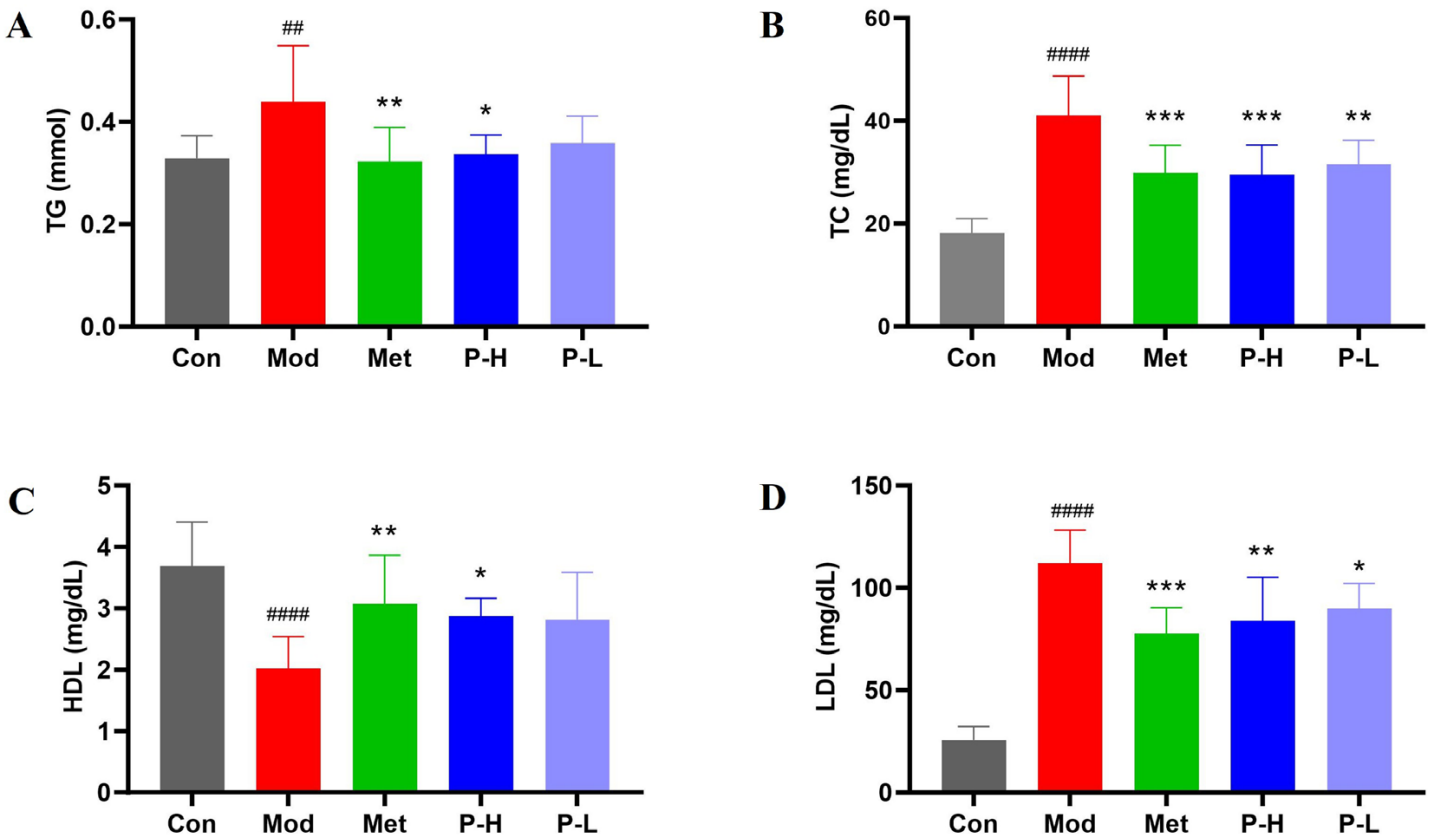
Fuc‐S ameliorates dyslipidemia in STZ/HFD‐induced diabetic mice. (A) TG, (B) TC, (C) HDL, and (D) LDL levels. Following T2DM induction (4‐week HFD + STZ [25 mg/kg i.p., 3 days]), hyperglycemic mice (fasting glucose ≥ 11 mmol/L, 7 days) were treated with Fuc‐S or Met for 5 weeks. Serum lipids were quantified using commercial kits (*n* = 8, mean ± SEM). ^##^ *p* < 0.01, ^####^ *p* < 0.001 versus Con; *p < 0.05, **p < 0.01, ***p < 0.001 versus Mod. Groups: Con (control), Mod (model), Met (metformin), P‐H/L (high/low‐dose Fuc‐S).

Diabetic dyslipidemia, characterized by elevated serum TG, TC, and LDL alongside reduced HDL, is a critical driver of cardiovascular complications. Our data revealed that Fuc‐S intervention significantly counteracted these perturbations, effectively restoring lipid profiles to near‐normal levels (Figure 2). This suggests that Fuc‐S exerts dual benefits in T2DM by concurrently modulating glucose and lipid metabolism.

### 3.3 Fuc‐S Attenuates Diabetes‐Induced Hepatic Injury

**FIGURE 3:**
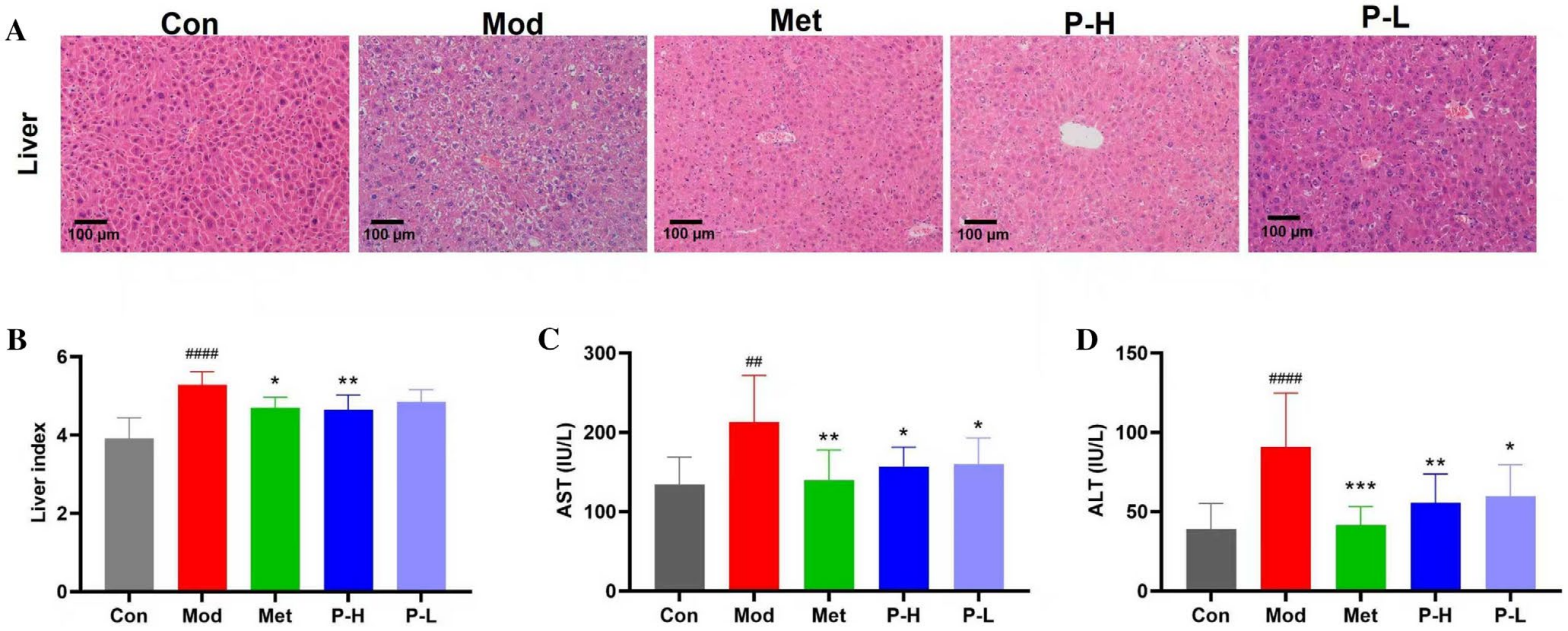
Fuc‐S ameliorates diabetes‐associated hepatic injury. (A) Representative H&E‐stained liver histology (x 200, scale bar = 100 μm). (B) Hepatosomatic index. (C) AST and (D) ALT serum levels. T2DM was induced by 4‐week HFD followed by STZ (25 mg/kg/day i.p., 3 days). Hyperglycemic mice (≥ 11 mmol/L, 7 days) were treated for 5 weeks with Fuc‐S or metformin. Liver and serum analyses were performed (*n* = 8, mean ± SEM). ^##^ *p* < 0.01, ^####^ *p* < 0.001 versus Con; *p < 0.05, **p < 0.01, ***p < 0.001 versus Mod. Groups: Con (control), Mod (model), Met (metformin), P‐H/L (high/low‐dose Fuc‐S).

Systematic evaluation of liver pathology demonstrated that Fuc‐S treatment mitigated hallmark features of diabetic hepatopathy. Histological analysis revealed a reduction in lipid droplet accumulation and steatosis in hepatocytes (Figure 3A), accompanied by normalized liver‐to‐body weight ratios (*p* < 0.05; Figure 3B). Concordantly, Fuc‐S significantly lowered serum AST and ALT levels—key indicators of hepatic damage—confirming its hepatoprotective efficacy (Figure 3C,D).

### 3.4 Fuc‐S Modulates Hepatic Antioxidant Defenses and Inflammatory Responses

**FIGURE 4:**
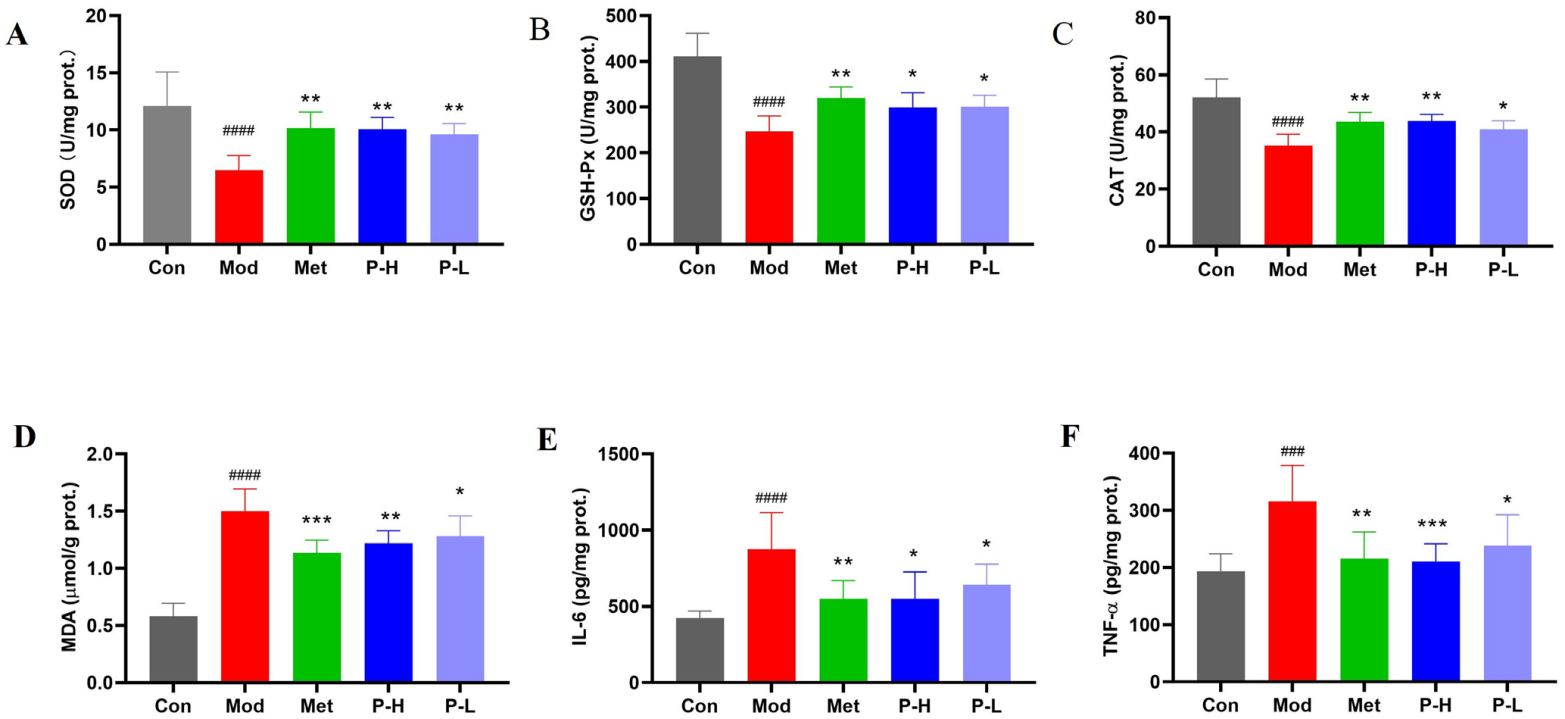
Fuc‐S improves hepatic antioxidant defense and reduces inflammation in diabetic mice. (A) SOD, (B) GSH‐Px, and (C) CAT activities; (D) MDA content; (E) IL‐6 and (F) TNF‐α levels. Following T2DM induction (4‐week HFD + STZ [25 mg/kg/day i.p., 3 days]), hyperglycemic mice (fasting glucose ≥ 11 mM, 7 days) received 5‐week treatment with Fuc‐S or metformin. Liver tissues were analyzed post‐treatment (*n* = 8, mean ± SEM). ^####^ *p* < 0.001 versus Con; *p < 0.05, **p < 0.01, ***p < 0.001 versus Mod. Groups: Con (control), Mod (model), Met (metformin), P‐H/L (high/low‐dose Fuc‐S).

Oxidative stress and chronic inflammation are pivotal interconnected drivers in the pathogenesis of diabetic liver injury. Mechanistic investigations revealed that Fuc‐S treatment effectively restored the hepatic redox balance. This was evidenced by a significant upregulation in the activities of key endogenous antioxidant enzymes, including SOD, CAT, and GSH‐Px. These enzymes constitute the primary cellular defense system against ROS, with SOD catalyzing the dismutation of superoxide radicals, and CAT and GSH‐Px eliminating hydrogen peroxide and lipid hydroperoxides, respectively (Figure 4A–C). Concurrently, Fuc‐S administration markedly suppressed lipid peroxidation, as indicated by a significant reduction in MDA, a well‐established biomarker of oxidative membrane damage (Figure 4D). The coordinated enhancement of the intrinsic antioxidant capacity and the attenuation of lipid peroxidation demonstrate that Fuc‐S mitigates oxidative stress in the diabetic liver. In parallel, Fuc‐S treatment led to substantial reductions in the hepatic levels of pro‐inflammatory cytokines, namely IL‐6 and TNF‐α (Figure 4E,F). The suppression of these pivotal inflammatory mediators further underscores the anti‐inflammatory efficacy of Fuc‐S. Collectively, these findings indicate that Fuc‐S alleviates diabetes‐associated liver injury not through a single action, but via coordinated antioxidant and anti‐inflammatory actions.

### 3.5 Fuc‐S Restructures Gut Microbial Ecology in Diabetic Mice

**FIGURE 5:**
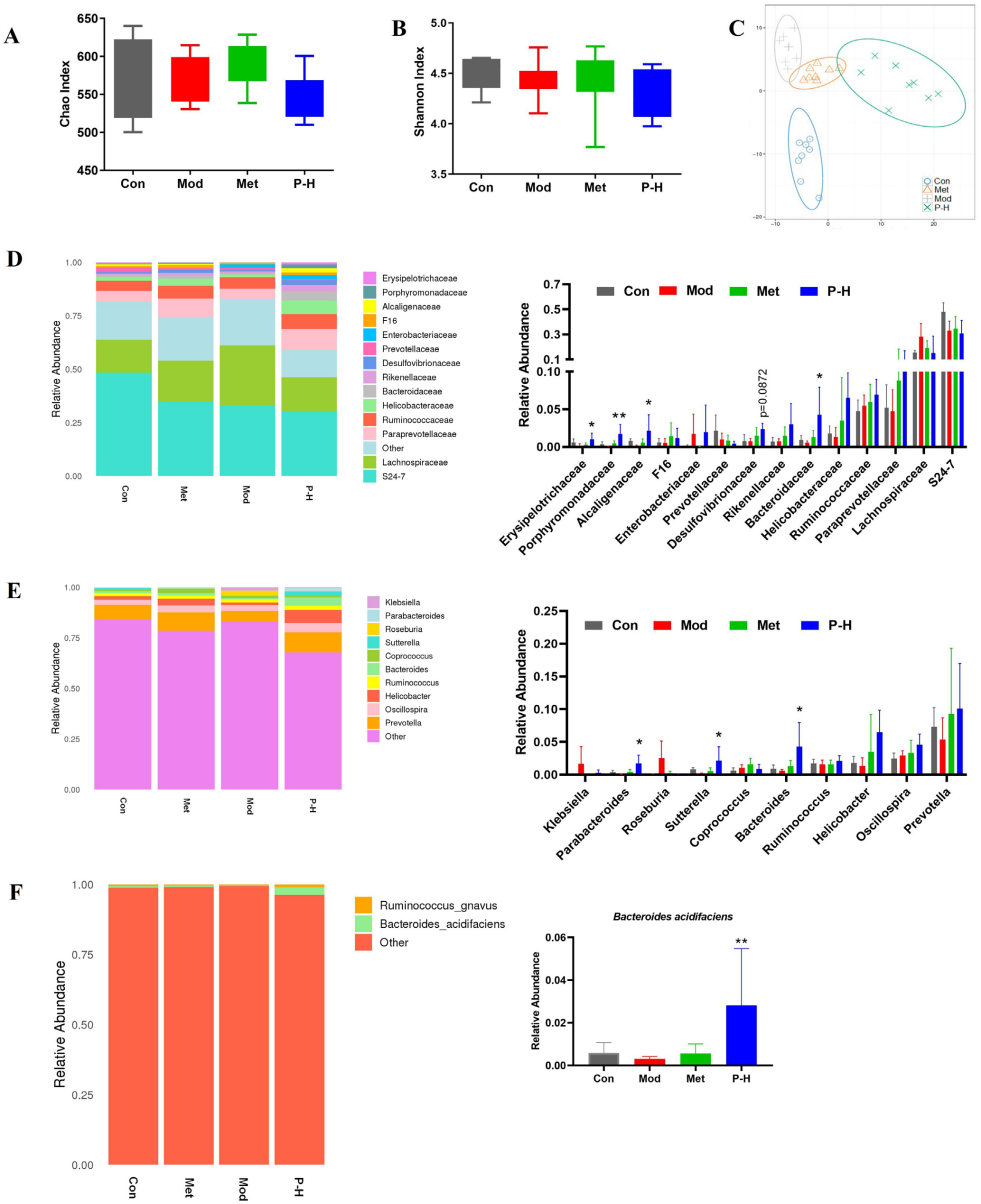
Effect of Fuc‐S on gut microbial ecology in STZ/HFD‐induced diabetic mice. (A) α‐diversity (Chao1 index), (B) microbial richness (Shannon index), and (C) β‐diversity (PCA score plot). (D) Family‐level, (E) genus‐level, and (F) species‐level taxonomic profiles. The T2DM model was induced by 4‐week HFD followed by STZ (25 mg/kg/day i.p., 3 days). Hyperglycemic mice (fasting glucose ≥ 11 mM, 7 days) were treated with Fuc‐S or metformin for 5 weeks. Fecal 16S rRNA sequencing was performed (*n* = 8, mean ± SEM). *p < 0.05, **p < 0.01 versus Mod group. Groups: Con (control), Mod (model), P‐H (high‐dose Fuc‐S).

High‐throughput sequencing revealed that Fuc‐S induced selective remodeling of the gut microbiota without altering overall α‐diversity (Chao1/Shannon indices; Figure 5A,B). PCA analysis confirmed distinct microbial community restructuring (Figure 5C), with notable enrichment of beneficial taxa such as *Bacteroidaceae* and *Desulfovibrionaceae* at the family level (Figure 5D). At finer taxonomic resolutions, Fuc‐S promoted the proliferation of probiotics including *Bacteroides* and *Sutterella* (genus level; Figure 5E), particularly elevating the abundance of the antidiabetic bacterium *Bacteroides acidifaciens* (species level; Figure 5F).

### 3.6 Fuc‐S Enhances Fecal SCFA Production

**FIGURE 6:**
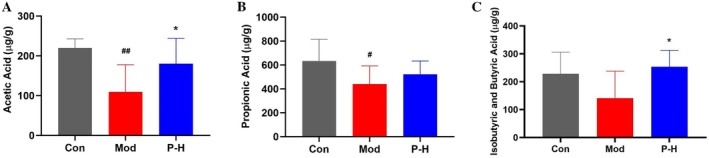
Fuc‐S enhances fecal SCFA production in STZ/HFD‐induced diabetic mice. (A) Acetate, (B) propionate, and (C) isobutyrate/butyrate levels. T2DM was induced by a 4‐week HFD and STZ (25 mg/kg/day i.p., 3 days). Following confirmed hyperglycemia (fasting glucose ≥ 11 mM, 7 days), mice received Fuc‐S or metformin for 5 weeks. Fecal SCFAs were quantified (*n* = 8, mean ± SEM). ^#^ *p* < 0.05, ^##^ *p* < 0.01 versus Con; *p < 0.05 versus Mod. Groups: Con (control), Mod (model), P‐H (high‐dose Fuc‐S).

The gut‐liver axis is integral to maintaining metabolic homeostasis, with SCFAs—microbial metabolites derived from dietary fiber—serving as pivotal mediators of this cross‐talk. As illustrated in Figure 6, the diabetic model group exhibited a significant decline in the levels of major physiologically active SCFAs, namely acetate, propionate, and butyrate/isobutyrate, compared to the normal control. Treatment with Fuc‐S notably reversed this trend, resulting in a marked upregulation of these SCFAs. The increases in acetate and butyrate/isobutyrate were particularly significant, showing statistically substantial differences (*p* < 0.05) relative to the untreated diabetic model. These results demonstrate that Fuc‐S can effectively restore the diabetes‐induced impairment of microbial SCFA production.

### 3.7 Fuc‐S Preserves Intestinal Barrier Integrity

**FIGURE 7:**
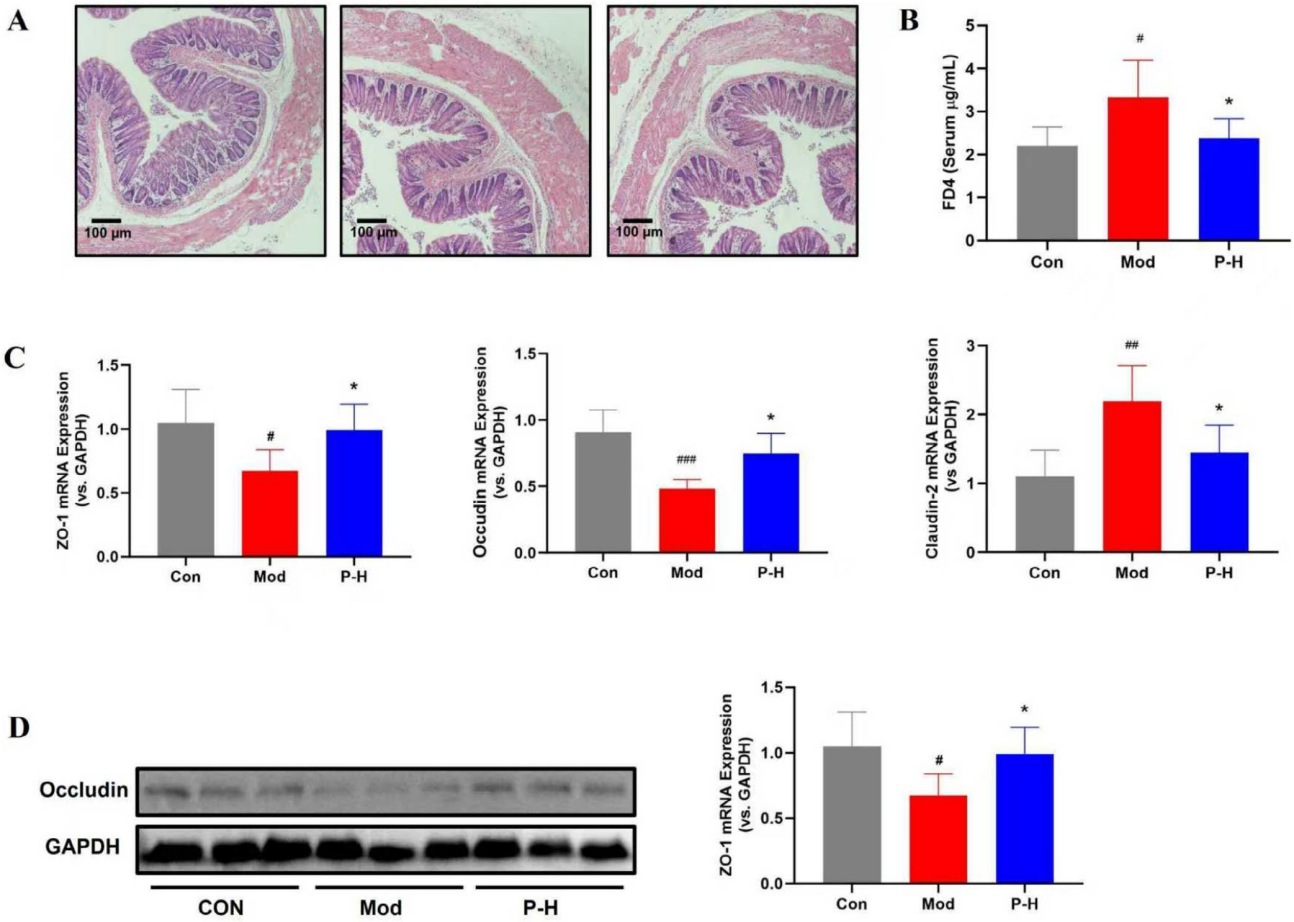
Fuc‐S Preserves Intestinal Barrier Integrity of STZ/HFD‐induced diabetic mice. (A) Representative H&E‐stained colon histology (× 200, scale bar = 100 μm). (B) Concentration of FITC‐Dextran in mouse serum. (C) mRNA levels of tight junction ZO‐1 and occludin and claudin‐2 (*n* = 8, mean ± SEM). (D) Protein expression of occludin (*n* = 3, mean ± SEM). T2DM was induced by 4‐week HFD and STZ (25 mg/kg/day i.p., 3 days). Following confirmed hyperglycemia (fasting glucose ≥ 11 mM, 7 days), mice received Fuc‐S for 5 weeks. Colon tissues were analyzed post‐treatment. ^#^ *p* < 0.05, ^##^ *p* < 0.01 versus Con; *p < 0.05 versus Mod. Groups: Con (control), Mod (model), P‐H (high‐dose Fuc‐S).

The integrity of the intestinal barrier is paramount in preventing the translocation of gut‐derived pathogens and endotoxins, a process implicated in the hepatic inflammation of diabetes. Given its SCFA‐promoting effects, we posited that Fuc‐S would directly strengthen intestinal barrier function. Our findings confirmed that Fuc‐S effectively ameliorated diabetes‐induced intestinal damage. Histological assessment revealed a clear restoration of colonic mucosal architecture, with attenuated edema, ulceration, and leukocyte infiltration. Functionally, this structural improvement was corroborated by a significant reduction in intestinal permeability, as evidenced by decreased serum levels of FITC‐dextran (FD4). Mechanistically, Fuc‐S orchestrated a favorable remodeling of the tight junction complex, significantly enhancing the expression of the sealing proteins occludin and ZO‐1 while suppressing the pore‐forming protein claudin‐2 (Figure 7).

## 4 Discussion

This study demonstrates that fucoidan Fuc‐S significantly improved glycemic control (reduced fasting glucose and enhanced glucose tolerance) and lipid profiles (decreased TG, TC, LDL‐C; increased HDL‐C). It also attenuated hepatic steatosis and lowered serum ALT/AST levels, demonstrating hepatoprotective effects. Mechanistically, Fuc‐S modulated gut microbiota (enriched *Bacteroides acidifaciens* ) and increased SCFA production (acetate, propionate, butyrate), thereby reducing hepatic oxidative stress (restored SOD/CAT/GSH‐Px activity; decreased MDA) and inflammation (lowered IL‐6/TNF‐α). These findings reveal Fuc‐S exerts multi‐target effects via the “gut microbiota‐SCFA‐liver” axis, supporting its potential as a therapeutic agent for T2DM and its complications.

T2DM‐associated liver injury typically progresses through stages of NAFLD, fibrosis, and potentially cirrhosis, with pathogenesis involving insulin resistance, oxidative stress, chronic low‐grade inflammation, and gut dysbiosis (Friedman et al. 2018; Tilg et al. 2020). Our histopathological findings (Figure 3A) confirmed characteristic hepatic vacuolation in diabetic mice, highlighting the central role of lipid metabolism dysregulation. This metabolic disturbance stems from three key insulin resistance‐mediated pathological changes: (1) enhanced peripheral lipolysis and subsequent free fatty acid (FFA) overflow to the liver (Fabbrini et al. 2008); (2) activation of hepatic de novo lipogenesis via SREBP‐1c pathway (Postic and Girard 2008); and (3) impaired VLDL secretion capacity (Adiels et al. 2005). Importantly, Fuc‐S not only reduced serum TG levels but also alleviated hepatic steatosis, suggesting its ability to modulate the dynamic balance of hepatic lipid influx, synthesis, and efflux.

The excessive hepatic lipid accumulation triggers mitochondrial dysfunction and reactive oxygen species (ROS) overproduction (Begriche et al. 2006; Chen et al. 2020). Our data showed significantly decreased antioxidant enzyme activities (SOD, CAT, GSH‐Px) and elevated MDA levels in diabetic mice (Figure 4), confirming oxidative stress as a central player in diabetic hepatopathy. ROS exacerbates liver injury through multiple mechanisms: direct oxidative damage to cellular components, activation of inflammatory pathways (consistent with our observed TNF‐α and IL‐6 elevation), and induction of endoplasmic reticulum stress and apoptosis (Ajoolabady et al. 2023; Kamata et al. 2005). The simultaneous improvement of both oxidative and inflammatory markers by Fuc‐S suggests its unique ability to break the vicious cycle of diabetic liver injury through synergistic antioxidant and anti‐inflammatory actions.

Emerging evidence positions gut dysbiosis as an initiator of T2DM‐associated liver injury (Le Roy et al. 2013; Qin et al. 2012). Our 16S rRNA sequencing results (Figure 5) revealed significant microbial imbalance in diabetic mice, characterized by reduced abundance of SCFA‐producing bacteria (e.g., *Bacteroides acidifaciens* )—a pattern consistent with clinical observations in T2DM patients. Gut microbiota disturbances impact hepatic physiology through: (1) compromised intestinal barrier function and subsequent LPS translocation activating Kupffer cells (Cani et al. 2007; Wiest et al. 2017); (2) altered bile acid metabolism affecting FXR signaling (Jiang et al. 2015; Sayin et al. 2013); and (3) diminished SCFA‐mediated anti‐inflammatory effects via G‐protein‐coupled receptors (Kimura et al. 2013; Rios‐Covian et al. 2016). The observed Fuc‐S‐induced preservation of intestinal barrier integrity (Figure 7) and increased SCFA levels substantiate its therapeutic mechanism via the gut‐liver axis. In addition, dysfunction of the intestinal barrier could lead to endotoxemia (Liang et al. 2025), which is a condition in which blood LPS levels are elevated. LPS is a component of the outer gut microbiota membrane. It plays a crucial role in the pathogenesis of diabetes‐associated liver injury. We will focus on the relationship between metabolic endotoxemia and T2DM‐associated liver injury in future studies.

Compared to conventional hypoglycemic agents, Fuc‐S exhibits unique multi‐system regulatory properties: (1) intestinal microbiota remodeling (enriching beneficial families like *Bacteroidaceae*); (2) metabolic modulation through SCFA elevation; and (3) direct hepatic protection via oxidative stress and inflammation mitigation. This comprehensive action mode enables simultaneous intervention at multiple pathological nodes of T2DM‐associated liver injury, potentially explaining why Fuc‐S outperformed metformin in ameliorating hepatic steatosis and inflammation (Figures 3, 4). The striking enrichment of *Bacteroides acidifaciens* (Figure 5F)—a species known to improve insulin resistance via succinate production and suppress gut inflammation—may represent a key mediator of Fuc‐S's effects, warranting further investigation.

## 5 Conclusion

The low‐molecular‐weight fucoidan fraction Fuc‐S demonstrates significant protective effects against T2DM‐induced liver injury. By modulating gut microbiota composition—particularly through enrichment of beneficial species like *Bacteroides acidifaciens* —Fuc‐S enhances the production of hepatoprotective SCFAs. These microbial and metabolic changes collectively improve hepatic oxidative stress, suppress pro‐inflammatory responses, and attenuate lipid accumulation. Our findings establish that Fuc‐S exerts multi‐target therapeutic effects through the “gut microbiota‐SCFAs‐liver” axis, offering a novel nutritional intervention strategy for managing T2DM and its hepatic complications.

## Author Contributions

**Yuedong Yang:** conceptualization and project administration. **Guangtao Zhang:** conceptualization, methodology, project administration, and supervision. **Jiao Peng:** conceptualization, methodology, funding acquisition, and supervision. **Bin Du:** investigation, formal analysis, methodology, and writing – original draft. **Caihong Cheng:** investigation. **Jinxiu Feng:** investigation. **Yue Liang:** investigation. **Wang Tao:** investigation. **Baojun Xu:** writing – review and editing. All authors have read and agreed to the published version of the manuscript.

## Funding

This work was kindly funded by the Natural Science Foundation of Guangdong Province‐ Provincial and Enterprise Joint Fund Project (2021A1515220012), Guangdong Provincial Hospital Association pharmaceutical research fund (2022YXKY09), and Hebei Agriculture Research System HBCT2024140208.

## Conflicts of Interest

The authors declare no conflicts of interest.

## Data Availability

All data supporting the findings of this study are available within the article.
